# Anabolic response to essential amino acid plus whey protein composition is greater than whey protein alone in young healthy adults

**DOI:** 10.1186/s12970-020-0340-5

**Published:** 2020-02-10

**Authors:** Sanghee Park, David D. Church, Gohar Azhar, Scott E. Schutzler, Arny A. Ferrando, Robert R. Wolfe

**Affiliations:** 1 Department of Geriatrics, Donald W. Reynolds Institute on Aging, Center for Translational Research in Aging & Longevity, University of Arkansas for Medical Sciences, 4301 West Markham Street, Slot 806, Little Rock, AR 72205-7199 USA; 2 Department of Geriatrics, Donald W. Reynolds Institute on Aging, University of Arkansas for Medical Sciences, 4301 W Markham Street, Slot 748, Little Rock, AR 72205-7199 USA

**Keywords:** Essential amino acids, Whey protein, Whole body anabolic response, Fractional synthetic rate, Leucine, Stable isotope

## Abstract

**Background:**

We have determined the acute response of protein kinetics to one or two servings (6.3 g and 12.6 g) of a proprietary composition containing free-form essential amino acids (EAA) (3.2 g EAA per serving) and whey protein (2.4 g per serving), as well as the response to consumption of a popular whey-based protein supplement (Gatorade Recover) (17 g; 12.6 g protein).

**Methods:**

Whole-body rates of protein synthesis, breakdown and net balance (taken to be the anabolic response) were determined using primed-constant infusions of ^2^H_5_-phenylalnine and ^2^H_2_-tyrosine. Muscle protein fractional synthetic rate (FSR) was also determined with the ^2^H_5_-phenylalanine tracer.

**Results:**

Plasma EAA levels increased following consumption of all beverages, with the greatest response in the high-dose EAA/protein composition. Similarly, the increase in net balance between whole-body protein synthesis and breakdown was greatest following consumption of the high-dose EAA/protein composition, while the low-dose EAA/protein composition and Gatorade Recover induced similar increases in net balance. When the net balance response was normalized for the total amount of product given, the high- and low-dose EAA/protein beverages were approximately 6- and 3-fold more anabolic than the Gatorade Recover, respectively. The greater anabolic response to the EAA/protein composition was due to greater increases in whole-body protein synthesis with both doses, and a markedly greater suppression of whole-body protein breakdown in the high-dose group. Muscle protein FSR after beverage consumption reflected changes in whole-body protein synthesis, with the larger EAA/protein dose significantly increasing FSR.

**Conclusion:**

We conclude that a composition of a balanced EAA formulation combined with whey protein is highly anabolic as compared to a whey protein-based recovery product, and that the response is dose-dependent.

**Trial registration:**

ClinicalTrials.gov Identifier: NCT03502941. This trial was registered on April 19, 2018.

## Background

Intact proteins are popular dietary supplements to increase lean body mass, or more specifically muscle mass, by stimulating protein synthesis. Supplementation of the diet with intact proteins may take the form of protein-fortified prepared foods, or as pure protein. Whey protein isolate is the most popular pure protein supplement. EAA are the primary “active” components of dietary protein. EAA cannot be produced in the body, but are primarily responsible for the stimulation of muscle protein synthesis [1]. Non-essential amino acid consumption, with or without concomitant EAA consumption, fails to affect protein synthesis in healthy, well-nourished volunteers, whether at rest [1, 2] or after exercise [3, 4]. The response of muscle protein synthesis following ingestion of a composition of free EAA is more than twice the response to consumption of a comparable dosage (g/g) of whey protein isolate [5]. The greater anabolic impact of free-form EAA can be attributed to the more rapid increase in plasma concentrations following ingestion of EAA, as well as the higher peak concentrations. In addition, EAA supplements can be formulated to address altered metabolic conditions, such as aging [6]. While dietary supplements of EAA have distinct advantages, isolated intact proteins, such as whey protein isolate, have potential advantages as well. The protein synthetic response to consumption of an isolated intact dietary protein is sustained over a longer time than the response to free-form EAA because of the slower absorption of the component amino acids in dietary protein [7]. In addition, peptides formed in the digestion of dietary proteins (particularly whey protein) have been proposed to have unique nutritional advantages [8–11]. Taste preferences may also favor intact protein compositions. Thus, the concept of a nutritional composition that combines the beneficial effects of both free-form EAA and isolated dietary protein is appealing.

Previous studies have added free leucine to whey protein isolate to amplify the synthetic response, with mixed results [12, 13]. The rationale of combining leucine with intact protein is that leucine can activate the molecular mechanisms involved in the initiation of protein synthesis, so that the tissue is “primed” for a greater response to the amino acids absorbed from the dietary protein. While the addition of free leucine may enhance the acute synthetic response to whey protein, an imbalance in the plasma concentrations of EAA will likely develop. The EAA with the lowest concentration relative to demand will limit the anabolic response, regardless of the extent of excess of the other EAA, including leucine. Consequently, maintaining a balance of EAA that is roughly proportionate to the demand for each EAA is important. For this reason, the idea of combining a balanced formulation of all of the EAA with an intact protein is appealing. A combination of a balanced formulation of EAA and a high-quality intact protein should provide the beneficial effect of a rapid and large increase in leucine concentration to activate the protein synthesis at a molecular level, while also providing sufficient other EAA to maintain a prolonged availability of all the necessary precursors for protein synthesis.

In this study we have determined the acute response of protein kinetics to two doses of a composition containing free EAA and whey protein, as well as the response to consumption of an amount of a popular whey-based protein supplement.

## Methods

### Subjects

We studied 16 healthy male and females. Subject demographics are shown in Table 1. Potential subjects reported to the Reynolds Institute on Aging for informed consent discussion. The protocol, as well as the risks and potential benefits of participation in the study, were fully explained to the subjects prior to obtaining written (witnessed) informed consent. The study was approved by the Institutional Review Board of the University of Arkansas for Medical Sciences. Once consent was obtained, a medical history was obtained and a blood sample was drawn for complete blood count. A physical examination and a DEXA scan were performed.

**Table 1:** Subject characteristics

	Low- & High-dose EAA/protein	Whey Protein
Subject number (Male/Female)	8 (3/5)	8 (4/4)
Age, yr	21.4 ± 0.5	26.9 ± 2.0
Body weight, kg	73.8 ± 4.8	76.2 ± 3.1
Body mass index, kg/m^2^	24.6 ± 0.8	25.7 ± 1.6
Lean body mass, kg	51.6 ± 4.9	49.5 ± 2.6
Fat mass, %	21.1 ± 2.2	24.8 ± 4.1

Values are mean ± SEM.

### Experimental design

We used a randomized, two-period, stable isotope infusion study: a 4.5 h basal fasted period and a 4 h post-meal period (total 8.5 h). The principal end-point was the total anabolic response, or net protein balance (whole body protein synthesis minus breakdown). The secondary end-point was muscle protein fractional synthetic rate over the 4 h following the intervention of beverage consumption. The plasma amino acid concentration response was also a secondary end-point. The study design consisted of two arms. Arm 1 consisted of a group of subjects who performed a randomized, single blind cross-over (two stable isotope studies) in which they consumed each of the two doses of the proprietary free-form EAA/protein study supplement (6.3 g and 12.6 g), with a ≥ one week washout period between stable isotope studies. Arm 2 consisted of a group of subjects (with similar gender composition) who participated in one stable isotope study during which they ingested 17.6 g of a product (Gatorade Recover) containing 12.6 g of whey protein.

### Stable isotope tracer protocol

We have quantified the anabolic response to consumption of each beverage by determining whole-body protein kinetics (protein synthesis, protein breakdown, and net protein balance (g protein × 240 min)). We also measured the FSR of muscle protein to gain more direct insight into the response of muscle protein.

**Fig. 1:**
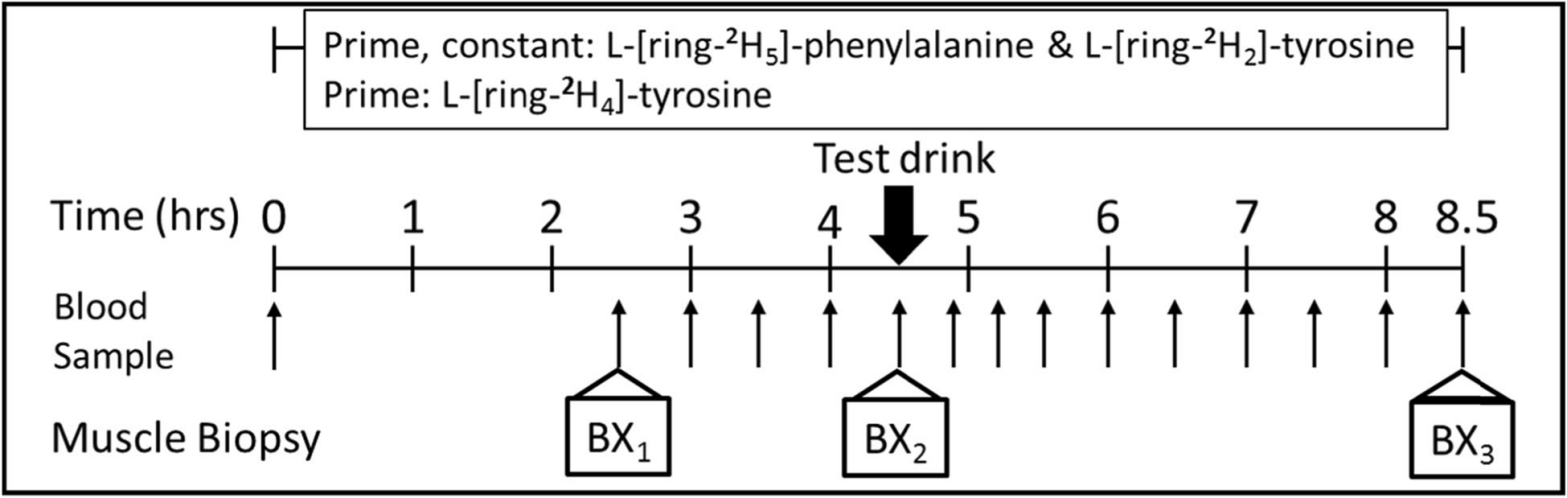
Tracer infusion protocol

Subjects reported to the Reynolds Institute on Aging having fasted overnight from 10:00 p.m. For subjects who participated in the dose-response study, the order of supplement dose was randomized. Intravenous catheters were inserted into a vein in each arm. One catheter was used to infuse the stable isotopes L-[ring-^2^H_5_] phenylalanine and L-[^2^H_2_] tyrosine) (Cambridge Isotope Laboratories, Andover, MA). The contralateral arm was used for blood sampling after warming the arm by means of a heated plastic box. After an initial blood sample was obtained, the primed-constant infusion of tracers was started as we have described previously [14, 15]. L-[ring-^2^H_5_] phenylalanine (prime, 4.60 μmol/kg; infusion rate, 3.92 μmol/kg/h) and L-[ring-^2^H_2_] tyrosine (prime, 0.95 μmol/kg; infusion rate, 1.57 μmol/kg/h) and a priming dose of L-[ring-^2^H_4_] tyrosine (0.33 μmol/kg) were started at time zero, and the tracer infusions maintained throughout the entire 8.5 h experimental period. Muscle biopsies were obtained after 2.5 h and 4.5 h in the basal state and at the end of the study (8.5 h) for determination of muscle protein FSR. The appropriate study supplement was served immediately after the second muscle biopsy was obtained (4.5 h). Subjects consumed each beverage within 5 min. The study protocol is shown schematically in Fig. 1. Blood samples were taken at 0, 150, 180, 210, 240, 270 min before consumption of a test supplement (fasted blood samples) and at 290, 310, 330, 360, 390, 420, 450, 480, and 510 min (fed blood samples) to measure tracer enrichment and plasma responses of essential amino acids. A total of 15 blood samples were taken (approximately 90 ml).

### Study products

A randomization list was used to determine which dosage of EAA/protein was tested first in the cross-over study (Arm 1). The study was blinded to the extent possible, given that different amounts of beverages were consumed. We considered the inability to completely blind the study of minimal concern, since neither the subject nor investigator had conscious control over their rates of protein synthesis and breakdown.

The composition of a single dose of each study product is shown in Table 2. Two doses of the EAA/protein beverage were given to provide 12.6 g. The EAA profile of the beverages was determined as described below for plasma free amino acids, with the additional step of hydrolyzing the whey protein in the Gatorade Recover beverage.

**Table 2:** Test product composition^a^

	EAA/protein study composition (6.3 g serving)	Gatorade Recover Whey Protein (17.9 g serving)
Free EAA	3.2 g	–
Creatine	0.5 g	–
Protein	2.4 g	12.6 g
Fat	0.0 g	1 g
Carbohydrate	0.0 g	2.6 g
Flavoring	0.2 g	1.7 g
	**% of total free EAA**	**% of protein-bound EAA**
Histidine	10	3
Isoleucine	10	12
Leucine	20	24
Lysine	19	18
Methionine	3	4
Phenylalanine	12	5
Threonine	16	19
Valine	10	16

^a^Subjects were given either one dose (6.3 g) or two doses (12.6 g) of the EAA/protein composition, or 17.9 g of the whey protein-based recovery product (Gatorade Recover)

### Analytic methods

Plasma samples were processed for determination of isotopic enrichment and amino acid concentrations as previously described [14, 16]. Tracer enrichments and plasma amino acid concentrations were determined by gas-chromatography mass spectrometry (GCMS: Models 7890A/5975; Agilent Technologies, Santa Clara, CA; LCMS:) and liquid-chromatography mass spectrometry (LCMS: QTrap 5500 MS;AB Sciex, Foster City, CA) as previously described [14, 15].

### Calculation of protein kinetics

Whole-body protein kinetics (protein synthesis, breakdown, and net balance) are expressed as changes from the basal, post-absorptive state to the fed state. Briefly, the calculation of protein breakdown in the post-absorptive state is based on the rate of appearance of phenylalanine (Phe) determined by traditional tracer dilution methodology, since Phe is not produced in the body. Protein synthesis is calculated as the difference between the rate of appearance of Phe and the irreversible loss of Phe, determined from the measured rate of hydroxylation of Phe to tyrosine (Tyr). Calculation of protein synthesis and net protein balance in the post-prandial state requires accounting for the contribution of the dietary Phe (exogenous appearance) to the total appearance of Phe in the blood. Exogenous appearance is equal to the amount of Phe absorbed minus whatever absorbed Phe is cleared irreversibly before reaching the peripheral blood where sampling occurs. We calculated the amount of Phe absorbed by multiplying the amount ingested by the true ileal digestibility (TID). TID was assumed to be 95.3%, for Gatorade Recover, assuming protein digestibility of the Gatorade Recover was the same as whey protein concentrate [17], and 98% for EAA/protein (100% for EAA, 95.3% for whey protein). The irreversible hydroxylation of absorbed Phe occurs in the liver [18] and is calculated by multiplying the measured fraction of Phe uptake hydroxylated by the amount of ingested Phe that was absorbed (ingested x TID). This calculation yields a value for the total 4 h post-prandial period. A 20% correction factor for the dilution of Phe tracer in the intracellular pool of the liver in the fasted state was applied to the calculation of the hydroxylation of Phe in the post-absorptive, but not post-prandial, state [19]. The total response of protein synthesis, protein breakdown, and net protein balance over the 4 hours after consumption of each of the beverages was calculated to minimize any uncertainties stemming from non-steady state calculations [20].

The FSR of muscle protein was calculated as described previously [20], using the plateau in Phe enrichment in the basal state and the enrichment of plasma Phe integrated over 4 h post ingestion of study product as the precursor enrichment in the post-prandial state.

### Statistical analysis

A two tailed paired student’s t-test was performed to compare differences between the low- and high-dose of EAA/protein drink (dose effect) and a one-way repeated measures ANOVA was performed to compare differences from the whey protein drink with respect to changes in whole-body protein synthesis, breakdown and net balance, FSR, and plasma amino acids (area under the curve for response over 4 h). In order to compare EAA/protein drinks and whey protein drink with respect to the time-course of responses of plasma amino acids, two-way repeated measures of ANOVA were performed, followed by a two tailed paired t-test (if necessary). Statistical significance was declared with the *p*-values of < 0.05. The statistical analysis was performed using IBM SPSS Statistic Package software version 24 for Window (SPSS, Chicago, IL).

## Results

### Plasma amino acid concentrations

**Fig. 2:**
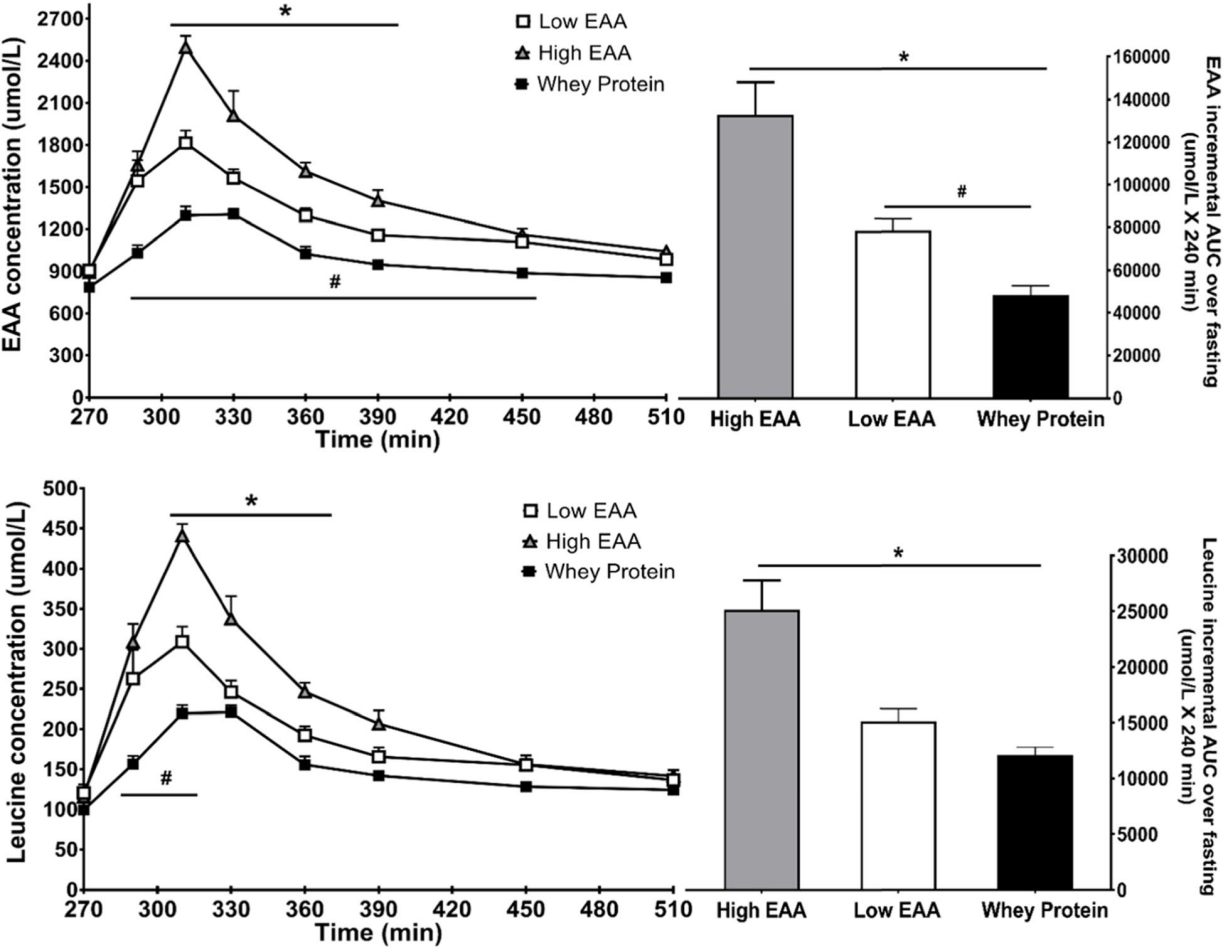
Total plasma essential amino acid (EAA) concentration (upper panel) and leucine concentration (lower panel) before and following consumption of one of two doses (6.3 g and 12.6 g) of the free EAA/protein composition or the whey protein product (17.9 g). Bar graphs on right represent the area under the curve for the response above baseline over the 4 h following consumption of each dose of free EAAs/protein and of whey protein. *Statistically different from High EAA, *p* < 0.05; ^#^Statistically different between Low EAA and whey protein, *p* < 0.05

Total plasma EAA concentration in the basal state and following beverage ingestion is shown in Fig. 2. The change in total EAA concentration following consumption of the EAA/protein beverage was directly related to the dose of study product. Both doses of the EAA/protein product caused significantly greater increases in EAA concentrations than Gatorade Recover. Plasma leucine increased to significantly higher values in both EAA/protein doses than Gatorade Recover, even though the amount of leucine (64 mg) ingested in the low-dose EAA/protein was less than the amount of leucine (108 mg) in the whey protein product (Fig. 2).

### Whole body anabolic response

**Fig. 3:**
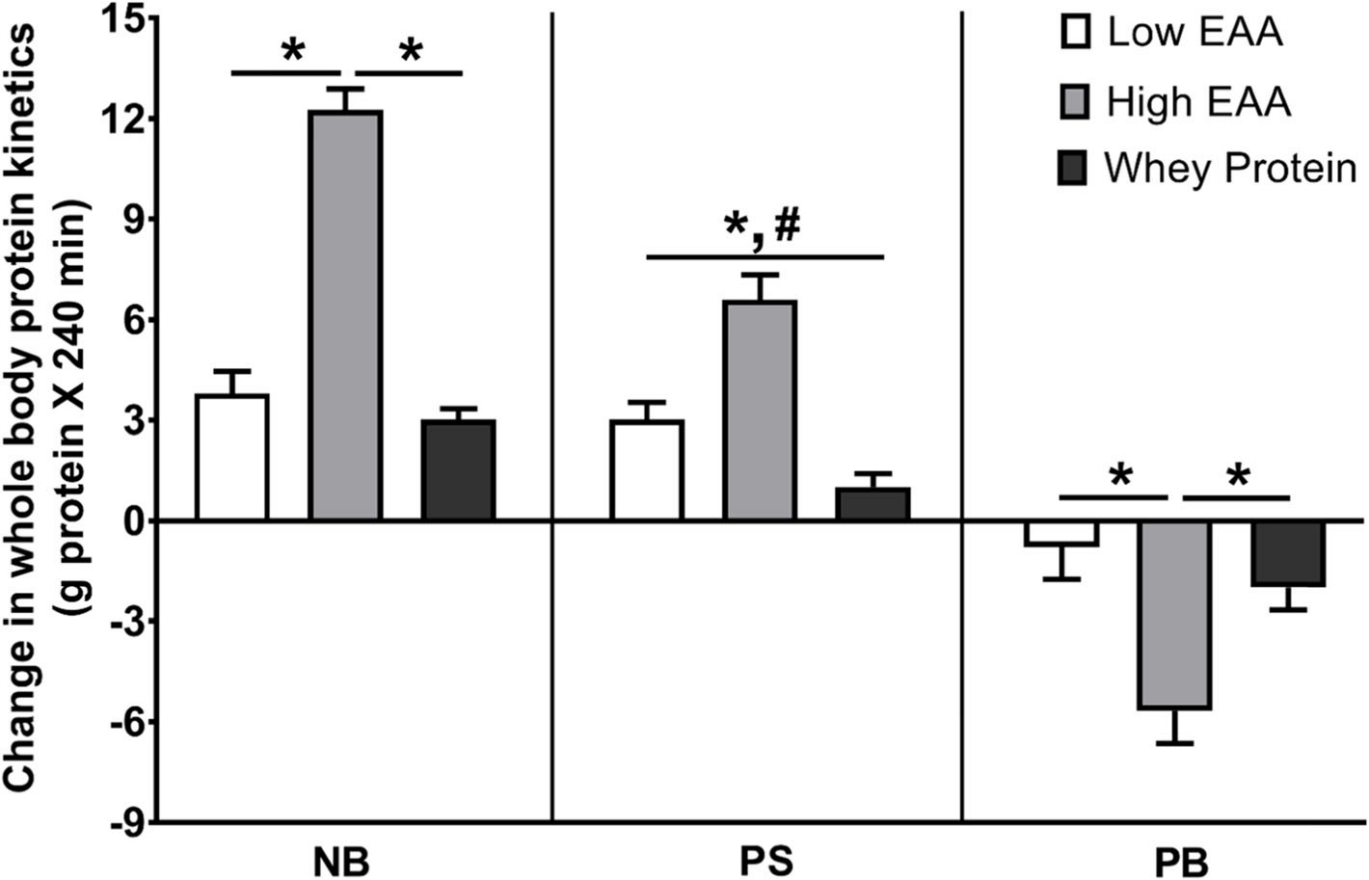
Changes from baseline of whole-body net protein balance (NB), protein synthesis (PS) and protein breakdown (PB) following consumption one of two doses of the free EAAs/protein composition (6.3 g and 12.6 g) and the whey protein product (17.9 g). *Statistically different from High EAA, *p* < 0.01; ^#^Statistically different between Low EAA and whey protein, *p* < 0.05

**Fig. 4:**
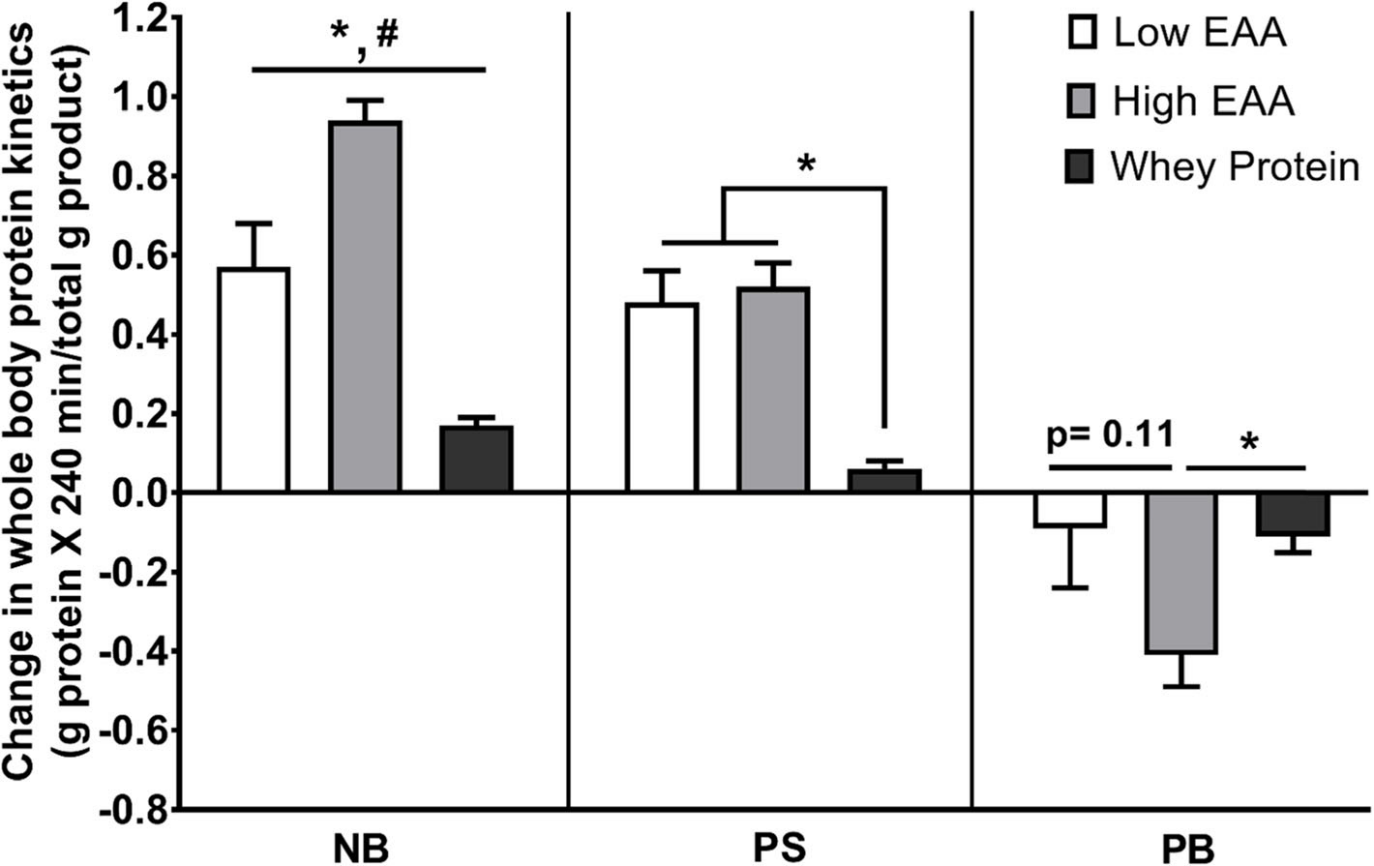
Changes from baseline of whole-body net protein balance (NB), protein synthesis (PS) and protein breakdown (PB) following consumption one of two doses of the free EAAs/protein composition (6.3 g and 12.6 g) and the whey protein product (17 g). Values are normalized for the amount of product consumed. *Statistically different from High EAA, *p* < 0.01; ^#^Statistically different between Low EAA and whey protein, *p* < 0.05

All three treatments caused an increase in net whole body protein balance (NB) (Fig. 3 and Additional file 1: Figure S1). The total gain in NB was greatest (11.8 ± 1.8 g protein for 240 min) in the group consuming 12.6 g of the free EAA/protein composition (statistically significantly greater than the other two groups, *p* < 0.01). The increase in NB in the group consuming 6.3 g of the free EAA/protein composition was similar to the group receiving 17.9 g Gatorade Recover. The greater increase in NB with the high-dose EAA/protein compositions was due to both greater increases in the rate of whole-body protein synthesis and greater suppression of whole-body protein breakdown as compared to the low-dose EAA/protein and Gatorade Recover. The increase in NB with the low-dose of EAA/protein composition was due to a mainly greater increase of protein synthesis while the increase in NB with Gatorade Recover was due to a combination of a modest increase in protein synthesis and suppression of protein breakdown as compared to the fasting state. When normalized for the amount of product given, the high- and low-EAA/protein composition was more than 6 and 3 times, respectively, as effective per gram of product given than whey protein, respectively (Fig. 4).

### Muscle fractional synthetic rate

**Fig. 5:**
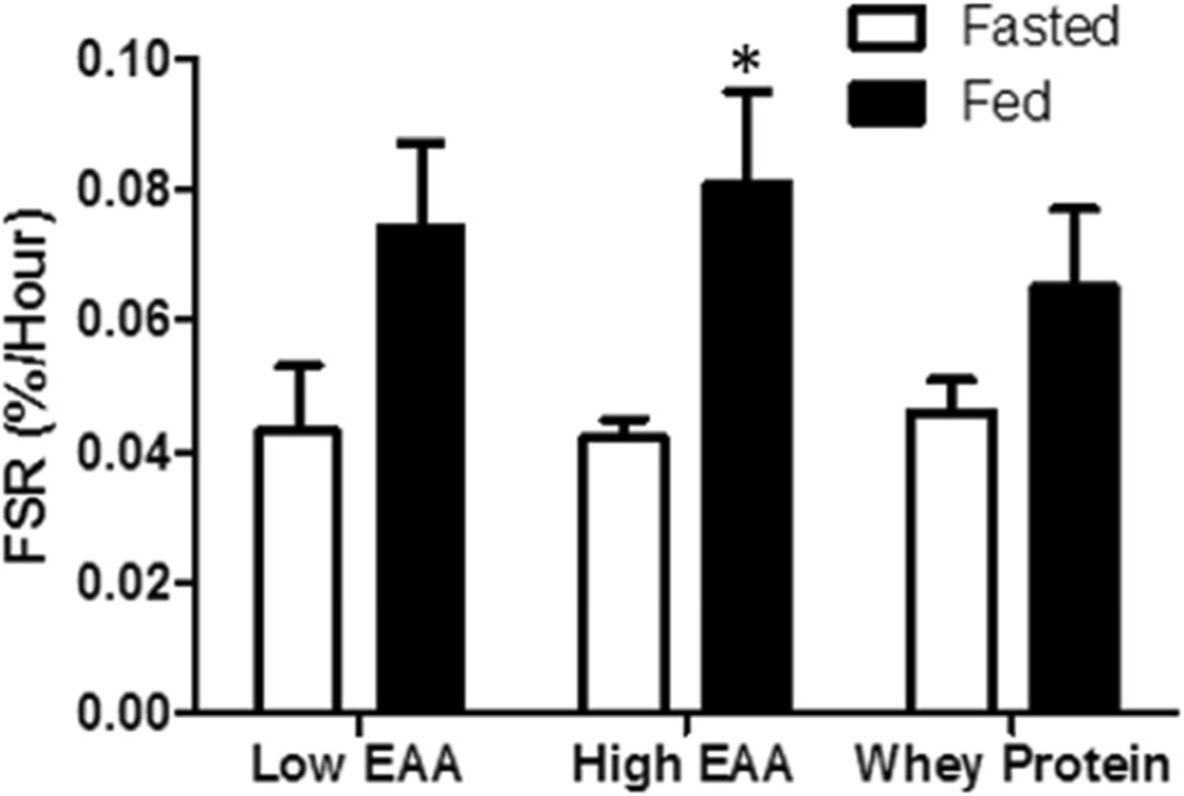
Muscle protein fractional synthesis rate (FSR) following consumption one of two doses of the free EAAs/protein composition (6.3 g and 12.6 g) and the whey protein product (17.9 g). *Statistically significant from fasted within treatment, *p* < 0.05

The pattern of response of muscle protein FSR was similar to that of whole body protein synthesis, but the magnitude of changes was smaller (Fig. 5). FSR increased significantly above the baseline value following consumption of both the low-dose and the high-dose free EAA/protein, but only the increase in the high-dose group (from 0.042 ± 0.003%/h to 0.081 ± 0.014%/h, *p* < 0.05) was statistically significant. The increase in FSR after consumption of the whey protein product was not statistically significant.

## Discussion

The principal finding of this study is that a combination of free EAA and whey protein is highly anabolic in healthy young volunteers. The anabolic response to the free EAA/protein composition was dose-dependent. Interestingly, the gain in NB following consumption of 12.6 g of free EAA plus whey protein was significantly greater than the response of NB to consumption of 6.3 g of the free EAA/protein product when normalized for the amount of product consumed (Fig. 4), due to a greater suppression of protein breakdown. The anabolic responses of either dose of free EAA/whey protein product was greater than the response to a whey protein-based commercial beverage when normalized to the amount consumed. When normalized for the amount of product consumed, the low dose free EAA/protein response of NB was approximately three- fold greater than the whey protein product, and the response of NB to the high dose of the free EAA/protein product was approximately six times greater than the response to the whey protein product.

The anabolic benefits of whey protein dietary supplements are well established, both in sedentary individuals as well as adjuncts to physical training (e.g., [21]. Similarly, the consumption of free EAA-based nutritional supplements are well-documented to stimulate muscle protein synthesis and net protein balance [5, 22], and sustained consumption improves physical function in older individuals [23]. The stimulation of muscle protein synthesis by consumption of less than 4 g of EAA has been reported to be as great as the response to consumption of 25 g dose of whey protein [24]. The enhanced anabolic effect of free EAA dietary supplements has been attributed by some to the activation of mTORC1 and related compounds involved in the initiation of protein synthesis [25]. Leucine in particular has been reported to play a key role in activating mTORC1, and thus the stimulation of muscle protein synthesis [26]. The notion that the addition of free leucine to a dose of dietary protein activates mTORC1, thereby amplifying the anabolic response to the amino acids in the protein, has been tested in previous studies [12, 13]. Results of studies in which free leucine has been added to dietary protein or to complete meals have been disappointing. In the circumstance of impaired anabolic responsiveness, such as occurs in cancer cachexia, the addition of leucine to a whey-based nutritional composition may enhance the anabolic response [27]. However, in healthy younger subjects, any beneficial effect of adding free leucine to intact protein is short-lived [25] or not detected [12, 28]. The problem with adding only leucine to dietary protein is that the availability of the other EAA becomes rate limiting. In particular, the plasma concentrations of the other branched chain amino acids (valine and isoleucine) fall below the fasting level when only extra leucine is added to intact protein [12].

The current study is the first of which we are aware in which a balanced formulation of free EAA has been combined with whey protein. The formulation differed from most EAA nutritional compositions in that leucine comprised only 20% of the free EAA. It has been postulated that the magnitude of anabolic response to dietary protein is determined by the increase in plasma leucine concentration, rather than to the amount of protein consumed [26]. In support of this perspective, EAA compositions designed for elderly individuals require a disproportionately high percentage of leucine to maximize the anabolic response than would be predicted from the composition of muscle protein [6]. However, disproportionately high leucine content in compositions designed to stimulate an anabolic response in younger heathy volunteers is not necessary [29]. Rather, in the current study the leucine content of the EAA/protein composition was based on the amount required to maintain a balance among all the protein synthetic precursors. By including only 20% of EAA as leucine, it was possible to increase the relative proportions of the other EAA, thereby providing all of the precursors necessary for synthesis of body proteins. Even with a low dose of free EAA comprised of only 20% leucine, the plasma leucine concentration rose almost 3-fold (Fig. 2), while the concentrations of the other EAA were increased in proportion to their requirements for muscle protein synthesis.

In addition to being able to produce a composition of exact proportions of EAA, free EAA have the advantage of being rapidly and completely absorbed [30]. The rapid peak response in plasma EAA is likely a key reason for their effectiveness [31]. On the other hand, the total duration of the response is limited, because just as the concentrations of EAA in the blood rise rapidly, they fall rapidly as well. For this reason, the composition tested in this study contains protein in addition to the EAA to prolong the anabolic response in the time after consumption.

Non-essential amino acids (NEAA) are not required for the acute anabolic response to EAA consumption [2–4]. This is because NEAA are normally produced in the body at fast enough rates to avoid deficiencies. On the other hand, studies performed in livestock suggest that maximal long-term animal growth and development is achieved with a balance of about 20–30% NEAA and 70–80% EAA [32]. The implication that NEAA availability can eventually become rate limiting for protein synthesis is supported by the fact that the NEAA, particularly alanine and glutamine, fall after consumption of a single dose of free-form EAA [33]. The addition of intact protein to a mixture of free-form EAA is the most efficient way to ensure an adequate amount of dietary NEAA to maximize long-term increases in lean body mass and physical function resulting from regular consumption. The action of peptides produced in the digestion of whey protein may have contributed to an interactive effect between free EAA and whey protein. Peptides of whey protein are reported to have a wide range of potential benefits (e.g., [8–11]), and amplifying the anabolic response to free EAA may be one such benefit. The current study design did not enable assessment of the role of peptides produced in the digestion of whey protein.

A comment about the relation between the whole-body protein and muscle protein FSR response is appropriate. Qualitatively the responses of muscle protein FSR were similar to the responses of whole-body protein synthesis with the three treatments. Further, the muscle FSR responses in the current study were generally in line with the results from comparable studies. For example, Churchward-Venne, et al., [7] reported that consumption of 1.5 g or 6 g of an EAA composition increased muscle FSR by 40 and 36%, respectively, as compared to a 50% increase following consumption 40 g of whey protein. The corresponding values in our study were 39 and 76% increases in FSR in response to 6.3 g and 12.6 g, respectively, of the free EAA/protein composition, and a 28% increase in response to the 12.6 g of whey protein in Gatorade Recover. However, in the current study the magnitude of the differences in whole-body net balance response between treatments was much greater than the differences in FSR, owing to a suppression of whole-body protein breakdown in addition to a greater stimulation of protein synthesis in the high-dose EAA/protein treatment. The two doses of the EAA/protein compositions resulted in increases in net protein balance of 3.6 ± 1.9 and 11.8 ± 1.8 g protein /4 h for the low- and high-dose free EAA/protein compositions, respectively, as compared to an increase of 3.0 ± 0.9 g for the Gatorade Recover. These results underscore the importance of quantifying both the rates of protein synthesis and breakdown when assessing the net anabolic response to a nutritional intervention.

The quantification of the response of whole-body net balance to nutrient consumption enabled the comparison of the amount of amino acids ± protein consumed with the net gain in body protein. The increase in body protein was approximately 24% of the amount of whey protein consumed with Gatorade Recover (Fig. 3). This percentage of net protein gain is consistent with the long-established relationship between N intake and N retention at levels of N intake above minimal requirements [34], and provides support for the quantitative validity of the whole-body protein model. In contrast to the response to whey protein, the gain in body protein was approximately 64 and 105% of the low-and high-doses of the free-form EAA/protein composition, respectively. The extraordinary increase of body protein in relation to the amount of amino acids in the free-form in the EAA/protein composition reflects the activation of the synthetic capacity by the rapid increase in EAA (including leucine) concentrations, the suppressive effects of a high-dose of EAA on protein breakdown [35–37], and the increased reutilization of endogenous NEAA to produce complete proteins.

It is appropriate to consider some of the advantages and limitations of quantifying the anabolic response by measurement of whole body protein synthesis and breakdown. Consideration of the response to nutrient ingestion at the whole body level is reasonable, since nutrients are consumed at the whole-body level. Importantly, whole-body protein turnover methodology enables the simultaneous determination of rates of protein synthesis and breakdown, and recent studies have highlighted the previously underappreciated role of protein breakdown in the anabolic response to protein intake [38]. Direct measurement of muscle protein FSR, on the other hand, provides information only on the protein synthetic response. Accurate measurement of net balance of muscle protein requires the invasive procedure of arterial and deep venous catheterization. Balanced against the advantages of whole-body protein kinetics, there are limitations. Calculated results reflect a pooling of the responses of all proteins in the body, and muscle protein may constitute as little as 25% of the total rate of whole body protein synthesis in some circumstances. Because the majority of whole body protein synthesis occurs elsewhere than the muscle, the rate of whole-body protein synthesis may not directly correspond to muscle protein FSR in some circumstances. However, with regard to the current study, the response of muscle FSR generally corresponded to the changes in whole-body protein synthesis, suggesting that at least some of the gain in net protein balance occurred in the muscle.

There are different methodological approaches to quantifying whole body protein synthetic and breakdown rates, all of which have advantages and limitations. We have recently discussed in detail the methodology used in the current study [39]. Importantly, we concluded that the necessary assumptions, while potentially contributing variability to the results, do not cause systematic over- or under-estimations of the calculated values. The validity of the whole-body methodology used in the current study is supported by comparison of the results to the results of other studies using different methodologies. As discussed above, there is a close relation between the net gain of body N following consumption of whey protein calculated by the tracer method and the value expected on the basis of previous N-balance studies. In addition, a key finding in the current study was that whole body protein breakdown was significantly suppressed with the highest dose of the EAA/protein composition. The suppressive effect of high concentrations of plasma amino acids on muscle protein breakdown in human subjects has been well-established for more than 20 years by arterial-venous balance studies [35–37].

## Conclusions

We conclude that there is an interactive effect between free EAA and whey protein that makes their combination highly anabolic in a dose dependent manner that exceeds the anabolic response to a whey-protein based supplement (Gatorade Recover) by approximately 3- and 6-fold for the low- and high-doses of free EAA/protein, respectively, when evaluated on a g/g basis.

## Supplementary information

**Additional file 1: Figure S1.** Whole body protein synthesis, Whole body protein breakdown.

## Data Availability

The datasets used or analyzed during the present study are available from the corresponding author on reasonable request.

## Abbreviations

EAA: Essential amino acids
FSR: Fractional synthetic rate
NB: Net whole body protein balance
NEAA: Non-essential amino acids
Phe: Phenylalanine
TID: True ileal digestibility
Tyr: Tyrosine
