# Supplementary material for: Anabolic response to essential amino acid plus whey protein composition is greater than whey protein alone in young healthy adults
Source: J Int Soc Sports Nutr. 2020 Feb 10;17:9. doi: 10.1186/s12970-020-0340-5 (PMC7011510; doi:10.1186/s12970-020-0340-5)
Supplement: Supplementary file 1 — Additional file 1: Figure S1. Whole body protein synthesis, Whole body protein breakdown. [file 12970_2020_340_MOESM1_ESM.pdf]

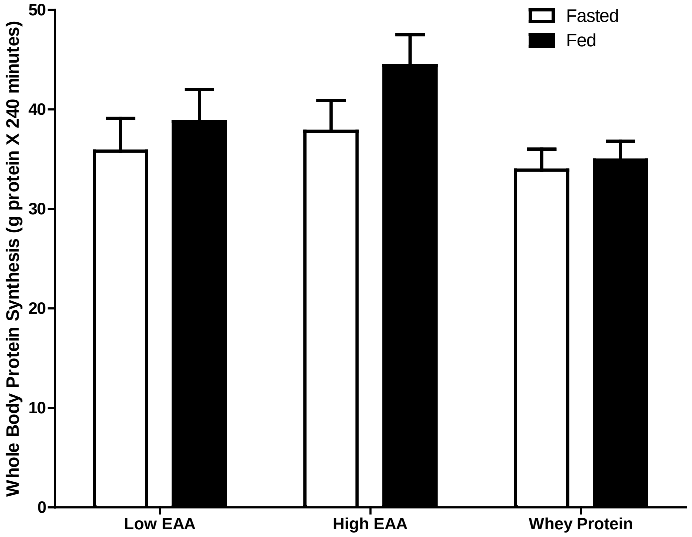

Absolute rates of whole body protein synthesis in the fasted and fed.

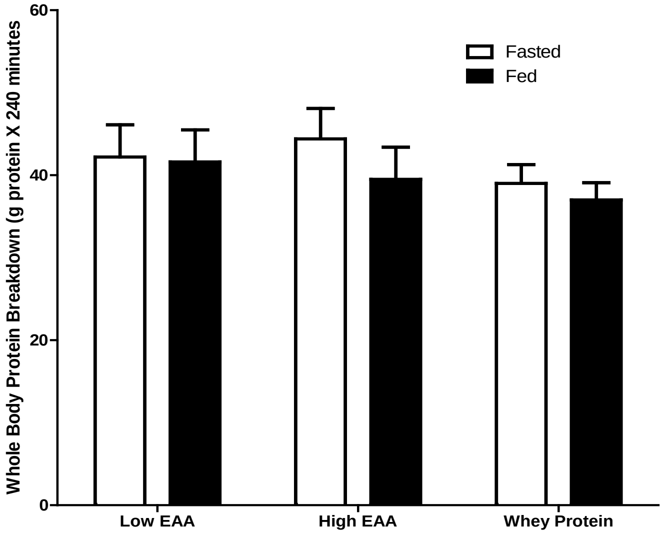

Absolute rates of whole body protein breakdown in the fasted and fed.
